# Development of a prognostic scoring model for predicting the survival of elderly patients with hepatocellular carcinoma

**DOI:** 10.7717/peerj.8497

**Published:** 2020-02-04

**Authors:** Sizhe Wan, Yuan Nie, Xuan Zhu

**Affiliations:** Department of Gastroenterology, The First Affiliated Hospital of Nanchang University, Nanchang, China

**Keywords:** HCC, Prognosis, Model, Survival, Elderly

## Abstract

**Background:**

The number of elderly hepatocellular carcinoma (HCC) patients is increasing, and precisely assessing of the prognosis of these patients is necessary. We developed a prognostic scoring model to predict survival in elderly HCC patients.

**Methods:**

We extracted data from 4,076 patients ≥65 years old from the Surveillance, Epidemiology, and End Results (SEER) database and randomly divided them into training and validation groups. Cox regression analysis was used to screen for meaningful independent prognostic factors. The receiver operating characteristic curve reflected the model’s discrimination power.

**Results:**

Age, race, American Joint Committee on Cancer stage, degree of tumour differentiation, tumour size, alpha-fetoprotein and tumour therapy were independent prognostic factors for survival in elderly HCC patients. We developed a prognostic scoring model based on the seven meaningful variables to predict survival in elderly HCC patients. The AUCs of the model were 0.805 (95% CI [0.788–0.821]) and 0.788 (95% CI [0.759–0.816]) in the training and validation groups, respectively. We divided the patients into low-risk groups and high-risk groups according to the optimal cut-off value. The Kaplan–Meier survival curve showed that in the training and validation groups, the survival rate of the low-risk group was significantly higher than that of the high-risk group (*P* < 0.001).

**Conclusion:**

Based on a large population, we constructed a prognostic scoring model for predicting survival in elderly HCC patients. The model may provide a reference for clinicians for preoperative and postoperative evaluations of elderly HCC patients.

## Introduction

Hepatocellular carcinoma (HCC) is one of the most common malignant tumours in the world, ranking 6th in global incidence and 4th among cancer-related deaths (Bray et al., 2018). Although the treatment of HCC has been significantly improved in recent years, the 5-year survival rate of patients is still at a low level, which brings great burden to individuals and society (Yang et al., 2019). As the ageing of the population intensifies, the number of elderly HCC patients also increases dramatically. Compared with young people, elderly cancer patients have higher morbidity and mortality after treatment (Turrentine et al., 2006; Ramesh, Jain & Audisio, 2005). Therefore, it is necessary to evaluate the prognosis of elderly patients with HCC.

The accurate prediction of prognosis in elderly patients with HCC is an important part of the management and treatment of HCC patients. Many HCC-related scoring systems have already been reported (Yang et al., 2011; Wong et al., 2014; Flemming et al., 2014), but there are few prognostic scoring models for the survival of elderly patients with HCC. A reasonable and effective survival prediction model can provide clinicians with effective guidance for preoperative evaluation and postoperative treatment of elderly HCC patients.

The Surveillance, Epidemiology, and End Results (SEER) database is a database based on the United States population that contains a wealth of relevant information for patients with different types of cancer, which provides a large amount of good information for us to study HCC. Some prediction models for tumours based on SEER database information have been reported (Henley et al., 2018; Melamed et al., 2018).

In this study, we included a large number of elderly HCC patients, explored factors related to survival and established a prognostic model to predict the survival of elderly HCC patients.

## Methods

### Patients

**Figure 1:**
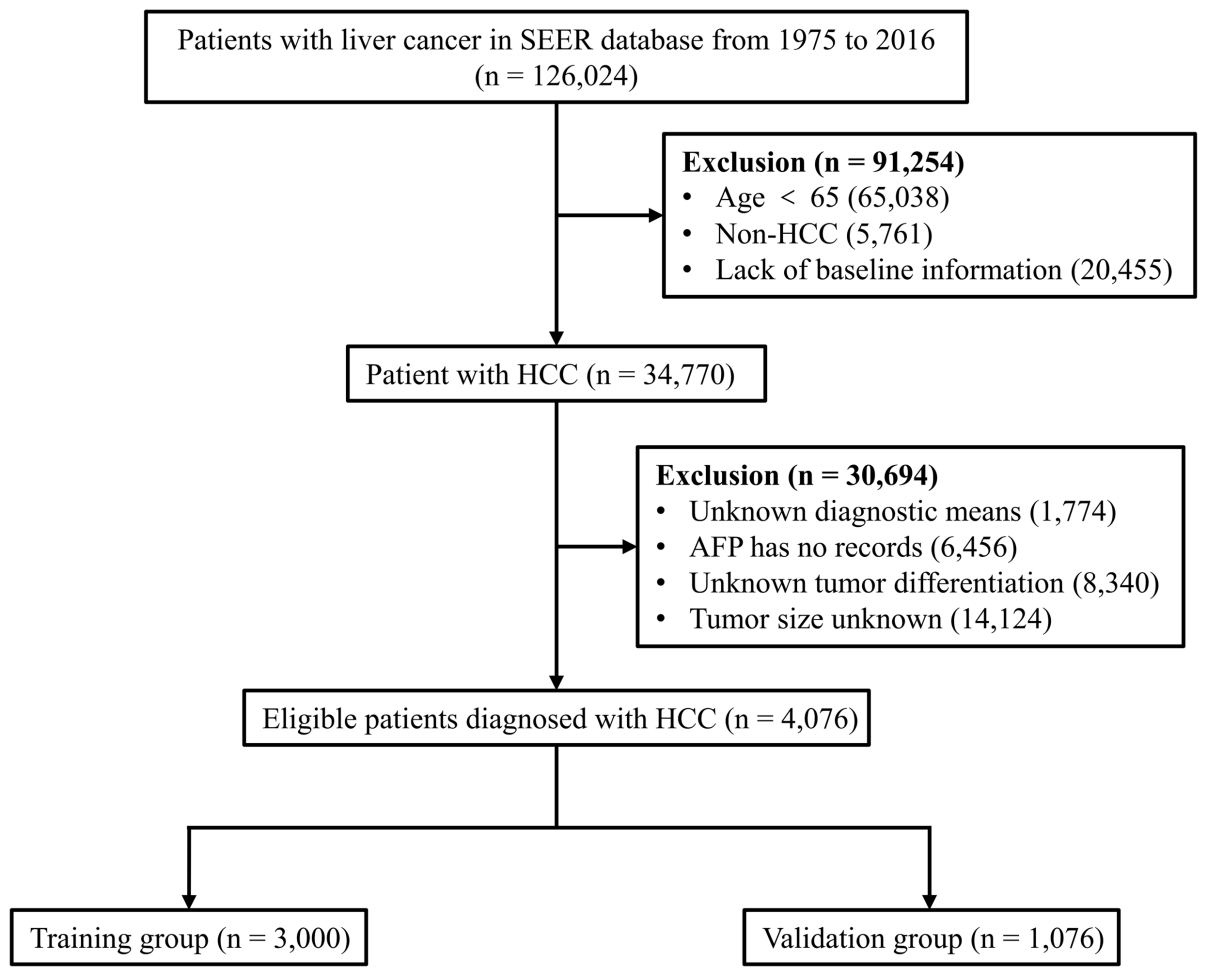
Flow diagram for selecting elderly HCC patients. SEER, Surveillance, Epidemiology and End Results database; AFP, alpha-fetoprotein; HCC, hepatocellular carcinoma.

Patients who met the following criteria were included in the study: (i) age ≥ 65 years; (ii) HCC patients, and (iii) complete patient information (baseline information, tumour-related information and outcomes). Patients younger than 65 years of age, those with non-HCC and those who lacked these data were excluded from the study. We extracted data from 126,024 liver cancer patients from 1975 to 2016 in the SEER database. We screened the data of this batch of patients several times. First, 65,038 patients younger than 65 years were identified, of which, 5,761 non-HCC patients with liver cancer (including cholangiocellular carcinoma, mixed cell liver cancer, etc.) and 20,455 patients with missing baseline characteristics (including age, sex, race, etc.) were excluded. Next, we eliminated 1,774 patients with unknown tumour diagnostic means, 6,456 patients with unknown alpha-fetoprotein (AFP), 8,340 patients with unknown tumour differentiation, and 14,124 patients with unknown tumour size. Finally, 4,076 eligible patients with complete information were included and randomly assigned to the training group (*n* = 3,000) or validation group (*n* = 1,076) (Fig. 1).

### Statistical analysis

Continuous variables were tested for normality, and if they follow a normal distribution, the mean ± standard deviation standard is provided, and Student’s *t* test was performed for comparisons between groups. The categorical variables are represented by counts and percentages, and the chi-square test and Fisher’s exact test were used to compare the groups.

Univariate Cox regression analysis was used for exploring potential prognostic factors. Factors with *P* values below 0.05 were included in the multivariate Cox regression by using the forward and maximal-likelihood ratio methods to screen out independent prognostic factors and to establish a Cox proportional hazards regression model. Survival was analysed by the Kaplan–Meier method, and the Kaplan–Meier curves of the subvariables were compared using the log-rank test. The receiver operating characteristic (ROC) curve was used to detect the discrimination power of the prognostic model. We determined the best cut-off value by the X-tile software. Statistical analyses were performed using SPSS 25.0. A two-side *P* value < 0.05 was considered statistically significant.

## Results

### Patient baseline characteristics

The clinical characteristics of the patients in the whole population, training group and validation group are listed in Table 1. Among the 4,076 HCC patients included in the total study, the average age was 73.65 ± 6.04 years old, with a male ratio of 71.4% and a white ethnicity ratio of 69.2%, indicating that the patients were mainly elderly. A total of 96.8% of patients were diagnosed with HCC via biopsy. In terms of tumour degree, most HCC patients (65%) were in stages 1 and 2 according to the 8th edition of American Joint Committee on Cancer (AJCC) (Amin et al., 2017) and had better differentiation (79.1%); only 6.1% of patients had tumour metastasis. There were 3,211 people with tumours greater than or equal to 3 cm and 2,555 people were AFP positive. More than half of the patients chose surgical treatment (58%). These baseline characteristics were similar in the training group of 3,000 patients and the validation group of 1,076 patients, and there was no significant difference.

**Table 1:** Baseline characteristics of elderly patients with HCC.

	All patients (*n* = 4,076)	Training group (*n* = 3,000)	Validation group (*n* = 1,076)	*P* value
Age (years^a^)	73.65 ± 6.04	73.55 ± 6.02	73.75 ± 6.07	0.572^b^
Sex, *n* (%)				
Male	2,929 (71.4)	2,137 (71.2)	772 (71.7)	0.749^c^
Female	1,167 (28.6)	863 (28.8)	304 (28.3)	
Race, *n* (%)				
White	2,820 (69.2)	2,084 (69.5)	736 (68.4)	0.359^c^
Black	400 (9.8)	301 (10.0)	99 (9.2)	
Other	856 (21.0)	615 (20.5)	241 (22.4)	
Diagnostic confirmation, *n* (%)				
Positive histology	3,947 (96.8)	2,914 (97.1)	1,033 (96.0)	0.069^c^
Non-positive histology	129 (3.2)	86 (2.9)	43 (4.0)	
AJCC stage, *n* (%)				
I	1,894 (46.5)	1,402 (46.7)	492 (45.7)	0.256^c^
II	755 (18.5)	571 (19.0)	184 (17.1)	
III	897 (22.0)	641 (21.3)	256 (23.8)	
IV	530 (13.0)	386 (12.9)	144 (13.4)	
Degree of tumour differentiation, *n* (%)				
I	1,305 (32.0)	965 (32.2)	340 (31.6)	0.920^c^
II	1,920 (47.1)	1,408 (46.9)	512 (47.6)	
III	790 (19.4)	584 (19.5)	206 (19.1)	
IV	61 (1.5)	43 (1.4)	18 (1.7)	
Tumour metastasis, *n* (%)				
Metastasis	247 (6.1)	175 (5.8)	72 (6.7)	0.311^c^
No metastasis	3,829 (93.9)	2,825 (94.2)	1,004 (93.3)	
Tumour size, *n* (%)				
<3 cm	865 (21.2)	643 (21.4)	222 (20.6)	0.581^c^
≥3 cm	3,211 (78.8)	2,357 (78.6)	854 (79.4)	
AFP, *n* (%)				
Positive	2,555 (62.7)	1,875 (62.5)	680 (63.2)	0.685^c^
Negative	1,521 (37.3)	1,125 (37.5)	396 (36.8)	
Tumour therapy, *n* (%)				
Non-surgery	2,366 (58.0)	1,723 (57.4)	643 (59.8)	0.078^c^
Local treatment	494 (12.1)	384 (12.8)	110 (10.2)	
Surgery	1,216 (29.8)	893 (29.8)	323 (30.0)	

**Notes:**

a The value represents the mean ± standard deviation.

b *t*-test between training group and validation group.

c Chi-square test between training group and validation group.

### Prognostic factors for the survival of elderly patients with HCC

We included all factors in the univariate regression analysis and identified nine variables that were significantly associated with survival, such as age, sex, race, etc. Next, we included these nine variables in the multivariate regression analysis and identified seven variables, age, race, AJCC stage, degree of tumour differentiation, tumour size, AFP and tumour therapy, that were independent prognostic factors for elderly patients with HCC (Table 2).

**Table 2:** Cox regression analyses of prognostic factors for the survival of elderly patients with HCC.

Variable	Univariable analysis			Multivariable analysis		
	HR	95% CI	*P* value	HR	95% CI	*P* value
Age (years)						
65–80	Reference			Reference		
>80	1.491	1.345–1.654	<0.001	1.223	1.101–1.358	<0.001
Sex						
Male	Reference					
Female	0.894	0.810–0.987	0.027			
Race						
Other	Reference			Reference		
White	1.412	1.254–1.589	<0.001	1.197	1.062–1.350	0.003
Black	1.485	1.250–1.764	<0.001	1.162	0.977–1.383	0.091
Diagnostic confirmation						
Positive histology	Reference					
Non-positive histology	1.235	0.973–1.568	0.083			
AJCC stage						
I	Reference			Reference		
II	1.082	0.951–1.231	0.234	1.071	0.940–1.220	0.305
III	2.458	2.197–2.751	<0.001	1.627	1.445–1.832	<0.001
IV	4.479	3.935–5.099	<0.001	2.534	2.210–2.906	<0.001
Degree of tumour differentiation						
I	Reference			Reference		
II	0.971	0.876–1.076	0.576	1.180	1.062–1.310	0.002
III	1.456	1.288–1.646	<0.001	1.651	1.451–1.877	<0.001
IV	1.625	1.138–2.320	0.007	1.773	1.237–2.541	0.002
Tumour metastasis						
No metastasis	Reference					
Metastasis	3.079	2.613–3.628	<0.001			
Tumour size						
<3 cm	Reference			Reference		
≥3 cm	1.982	1.756–2.238	<0.001	1.336	1.170–1.525	<0.001
AFP						
Negative	Reference			Reference		
Positive	1.556	1.415–1.710	<0.001	1.258	1.140–1.389	<0.001
Tumour therapy						
Surgery	Reference			Reference		
Local treatment	1.553	1.301–1.853	<0.001	1.935	1.611–2.325	<0.001
Non-surgery	4.824	4.265–5.457	<0.001	4.160	3.649–4.742	<0.001

**Note:**

HR, hazard ratio; 95% CI, 95% confidence interval; AJCC, American Joint Committee on Cancer; AFP, alpha-fetoprotein.

**Figure 2:**
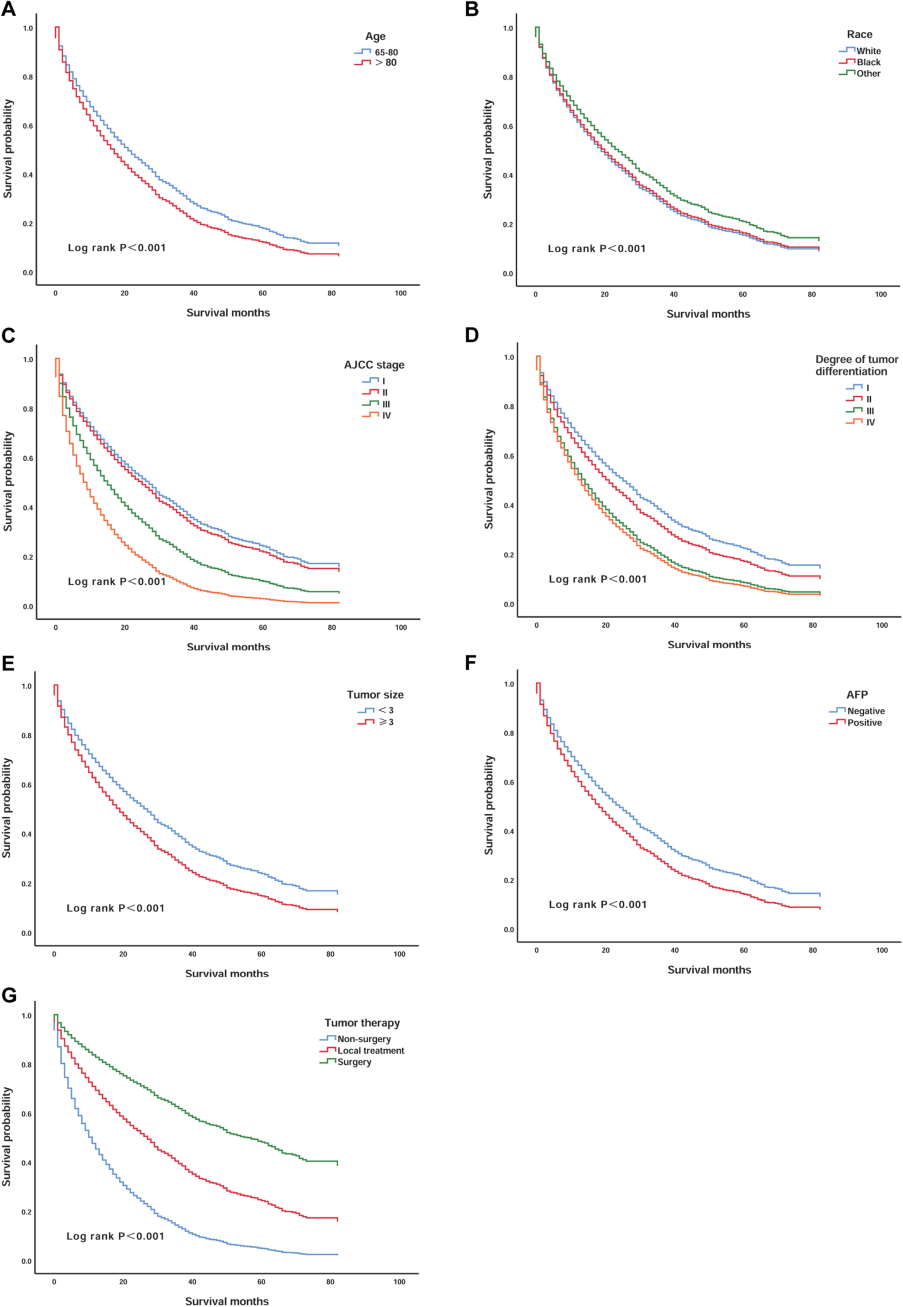
Kaplan–Meier survival curves of elderly patients with HCC stratified by age, race, AJCC stage, degree of tumour differentiation, tumour size, AFP, and tumour therapy. (A) According to age; (B) according to race; (C) according to AJCC stage; (D) according to the degree of tumour differentiation; (E) according to the tumour size; (F) according to AFP and (G) according to tumour therapy.

We validated the correlations between survival rate and the seven independent prognostic factors that were identified. Patients over 80 years of age had lower survival than those aged 65–80 years (*P* < 0.001) (Fig. 2A). White patients had worse survival than patients of other races (*P* < 0.001) (Fig. 2B). In addition, patients with AJCC stages III and IV and III and IV degrees of differentiation had lower survival (*P* < 0.001) (Figs. 2C and 2D). Patients with tumours larger than 3 cm that were AFP positive also had worse survival (*P* < 0.001) (Figs. 2E and 2F). However, patients who had undergone surgery had a significant increase in survival compared to the survival of patients who underwent other treatments (*P* < 0.001) (Fig. 2G).

### Development of the prognostic scoring model

**Figure 3:**
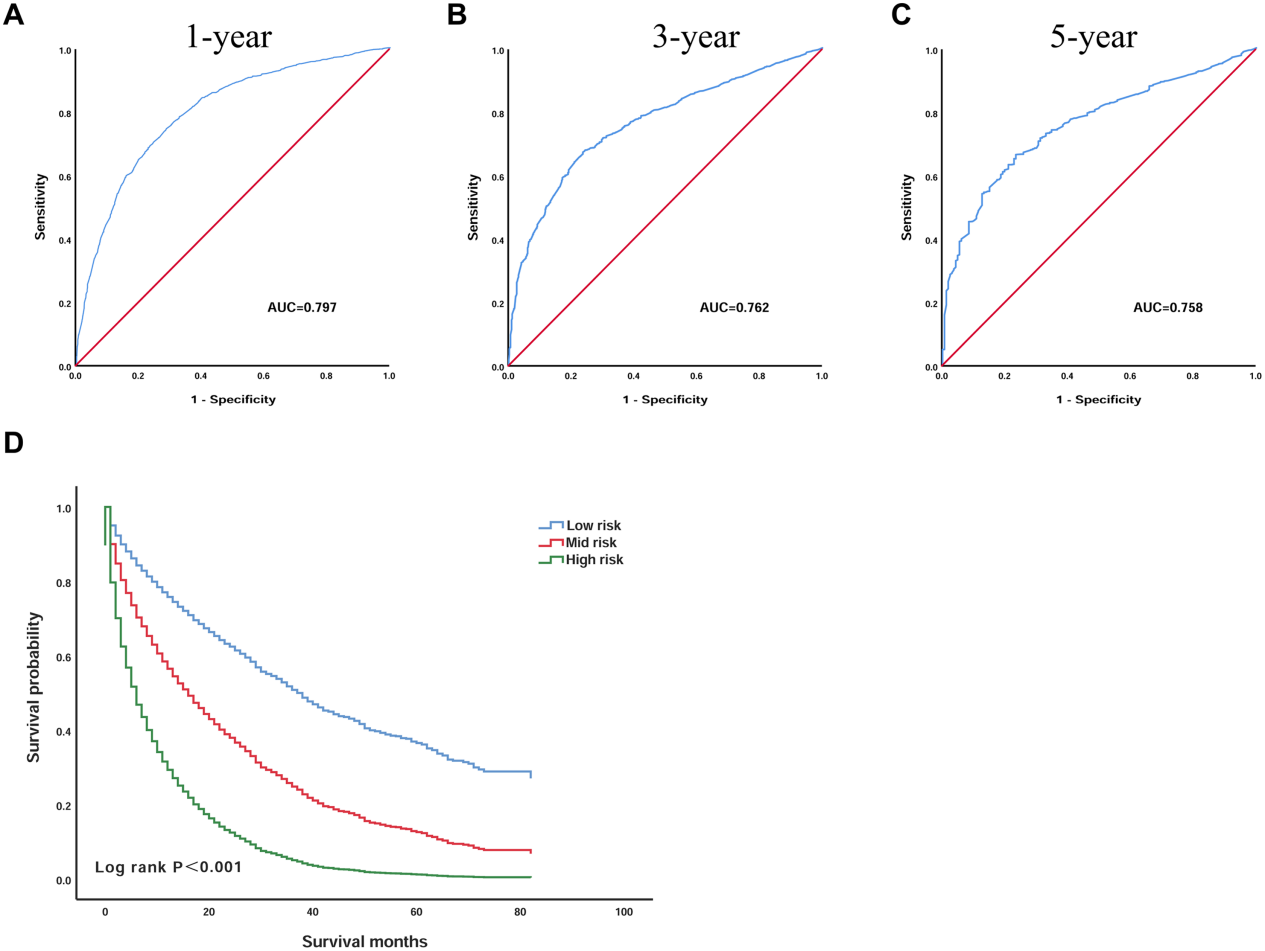
The prognostic scoring model has a good prognostic performance in predicting the overall survival of elderly patients with HCC. (A) ROC curve predicting 1-year survival in the training group. (B) ROC curve predicting 3-year survival in the training group. (C) ROC curve predicting 5-year survival in the training group. (D) Kaplan–Meier survival curves of the training group. ROC, receiver operating characteristic; AUC, area under the ROC curve.

Based on the seven independent prognostic factors selected by Cox’s proportional hazard regression analysis, we established a scoring model. The prognostic scoring model was as follows: overall survival (OS) = 0.196 × age − 0.083 × race + 0.241 × grade + 0.295 × AJCC − 0.731 × surgery + 0.319 × size + 0.225 × AFP. The area under the ROC curves (AUC) of the 1, 3 and 5 year survival probabilities were 0.797 (95% CI [0.781–0.813]), 0.762 (95% CI [0.742–0.782]), and 0.758 (95% CI [0.727–0.789]), respectively, indicating that the model has good discriminatory power (Figs. 3A–3C).

Then, by using X-tile software, the optimal cut-off value was selected as 1.3 and 2.6. Patients with an OS below 1.3 were included in the low-risk group (*n* = 1,215), patients with an OS greater than 1.3 and less than 2.6 were included in the mid-risk group (*n* = 1,390), and patients with an OS greater than 2.6 were included in the high-risk group (*n* = 398). The Kaplan–Meier curve was used to analyse the survival of the three groups (Fig. 3D). The OS of the low-risk group was significantly higher than that of the high-risk group, and the difference was statistically significant (*P* < 0.001). The 3 year survival rate was 34.5% and the 5 year survival rate was 11.0% in the low-risk group. However, the 3 year survival rate was 7.1% and the 5 year survival rate was 1.9% in the high-risk group, indicating that the scoring model has good practicability.

### Validation of the prognostic scoring model

**Figure 4:**
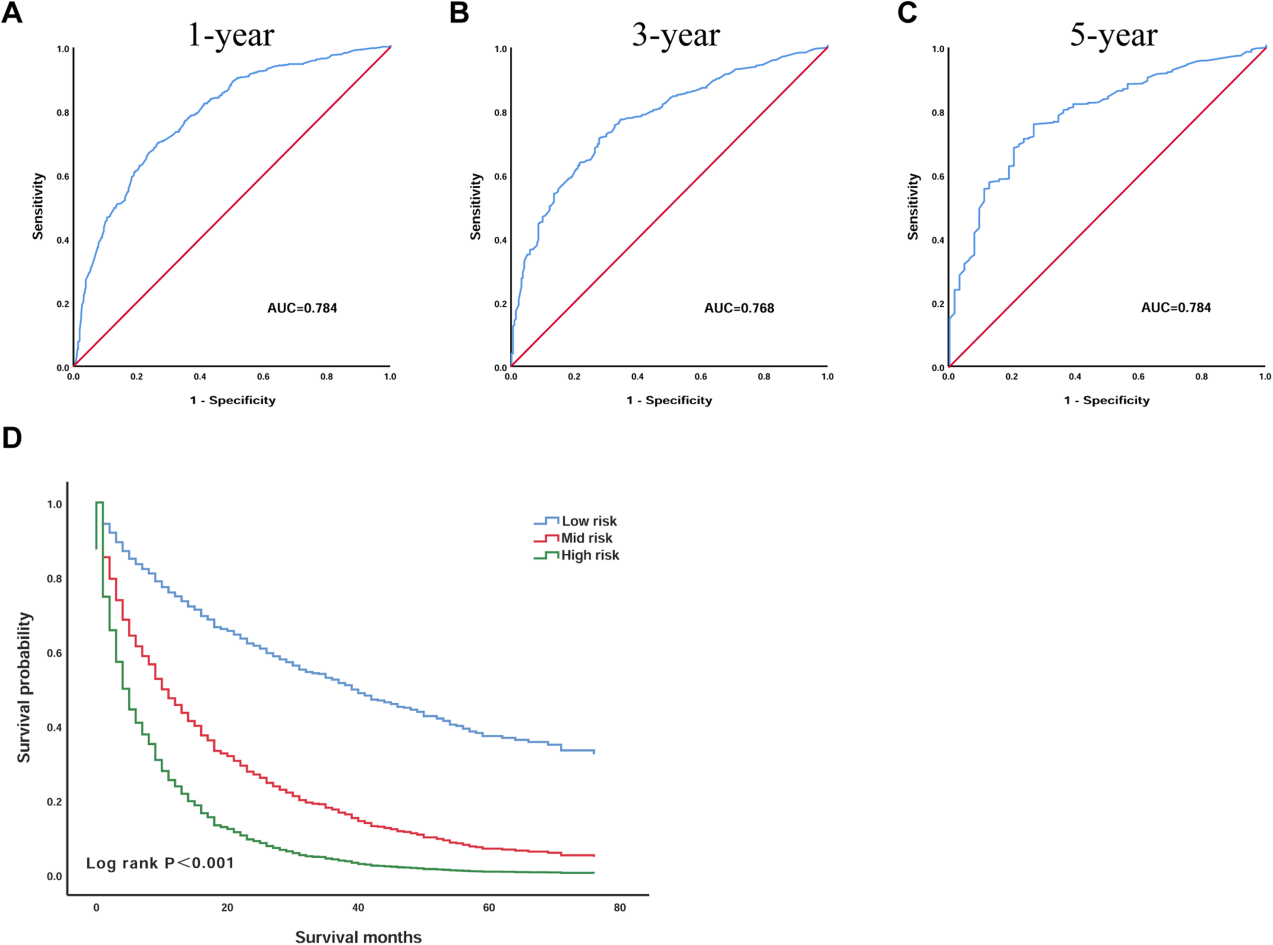
The prognostic scoring model had good prognostic performance for the overall survival of elderly patients with HCC in the validation group. (A) ROC curve predicting 1-year survival in the validation group. (B) ROC curve predicting 3-year survival in the validation group. (C) ROC curve predicting 5-year survival in the validation group. (D) Kaplan–Meier survival curves of the validation group. ROC, receiver operating characteristic; AUC, areas under the ROC curve.

To further confirm the reliability of the model, we validated the model in the validation group of patients. We plotted the ROC curves of the 1, 3 and 5 year survival probabilities in the validation group, with AUC of 0.784 (95% CI [0.757–0.811]), 0.768 (95% CI [0.735–0.800]), 0.784 (95% CI [0.732–0.836]), respectively (Figs. 4A–4C). According to the optimal cut-off value, we divided the 1,076 validation group patients: patients with an OS less than 1.3 into the low-risk group (*n* = 400), patients with an OS greater than 1.3 and less than 2.6 were included in the mid-risk group (*n* = 541), and those with an OS greater than 2.6 into the high-risk group (*n* = 135). Kaplan–Meier analysis suggested that the survival of the low-risk group was significantly higher than that of the high-risk group (*P* < 0.001) (Fig. 4D). In the low-risk group, the 3 year survival was 37.2% and the 5 year survival was 11.9%. In the high-risk group, the survival rate was lower; the 3 year survival rate was only 9.3%, and the 5 year survival rate was only 1.9%.

## Discussion

In this study, to better assess the survival of elderly HCC patients, we established a prognostic scoring model based on a large number of patients. In the training group and the validation group, a series of evaluations were performed on the discrimination power and practicability of the prognostic model.

Considering the increasing number of elderly cancer patients, this group of patients often has limited treatment options and poor prognosis. Therefore, it is very important to predict the prognosis of elderly patients with HCC. This prediction can provide a reasonable reference for clinicians to perform preoperative and postoperative treatments.

Through the Cox proportional hazards regression analysis, we selected seven independent predictors, age, race, AJCC stage, degree of tumour differentiation, tumour size, AFP and tumour therapy, and included them in the prognostic scoring model. Most of these included variables have been confirmed to be closely related to HCC. With the increasing number of elderly patients, the complexity of older patients, including the existence of multiple comorbidities, functional decline and cognitive impairment, and the competing risks of death and inadequate treatment, have made it difficult to predict the prognosis of elderly HCC patients (DeSantis et al., 2019). Dyson et al. (2014) reported that the mortality of elderly HCC patients is increasing annually. Kim et al. (2013) also confirmed that the poor prognosis of patients with HCC after percutaneous radiofrequency ablation is related to old age. A retrospective study in Japan found that age is an independent risk factor for the development of HCC in non-alcoholic fatty liver disease (NAFLD) patients (Kawamura et al., 2012). Our study also found age as a predictor of the prognosis of HCC patients.

We found that race is an important factor for the prognosis of elderly patients with HCC and that other ethnic groups, including American Indian/Alaska Native and Asian/Pacific Islander, have a higher survival rate than blacks and whites. In recent years, it has been reported that among all HCC patients, Asians or Pacific Islanders have the highest 5 year survival rate, followed by whites, while blacks have the lowest 5 year survival rate (Ha et al., 2016; Altekruse et al., 2012). Even in the Asian population, the survival rates of HCC patients from different Asian countries is significantly different (Pham et al., 2018). This shows that ethnicity is closely related to HCC prognosis.

The degree of tumour differentiation often affects the prognosis and choice of treatment options for cancer patients. Early studies have shown that the degree of tumour differentiation can be used as an independent risk factor for early HCC patients, and patients with better HCC differentiation have better prognosis (Nathan et al., 2009). A large randomised controlled trial (RCT) confirmed that tumour differentiation was a significant factor for local recurrence, OS, and cancer-free survival in HCC patients undergoing percutaneous radiofrequency thermal ablation, percutaneous ethanol injection, and percutaneous acetic acid injection (Lin et al., 2005). In addition, AJCC staging, or tumour-lymph node-metastasis staging, is also an important criterion for the current staging of cancer patients and is widely considered to be the standard approach for predicting prognosis in most solid tumour systems (Leung et al., 2002). This approach is consistent with our results; that is, tumour differentiation and AJCC staging are important prognostic factors in elderly HCC patients, and the survival of patients with poor differentiation and AJCC ≥ 3 stage is significantly lower than that of patients with high differentiation and AJCC < 3 stage.

HCC cases in which the maximum diameter of a single cancer nodule does not exceed 3 cm or the sum of the diameters of two cancer nodules does not exceed 3 cm is called small HCC. The HCC diameter value ‘3 cm or less (≤3 cm)’ has an important impact on the treatment options in the Barcelona Clinic Liver Cancer (BCLC) system and the third Japanese Society of Liver Diseases (JSH)-HCC guidelines (Kokudo et al., 2015). In the BCLC system, HCC ≤ 3 cm is considered to be ‘early’, and the cure rate is high. This finding may be based on the concept that HCC ≤ 3 cm is uniform and the malignant potential is low (Yamashita et al., 2018). A cohort study of 202 patients also confirmed our conclusion that patients with HCC with tumours smaller than 3 cm had longer survival after treatment (Camma et al., 2005). Llovet, Bru & Bruix (1999) also showed that patients with tumour nodules ≥3 cm have worse therapeutic outcomes and prognoses. Therefore, we classified elderly liver cancer patients with 3 cm as the standard and found that tumour size is closely related to patient survival.

AFP is an important indicator for screening liver cancer and prognosis. Bruix et al. (2017) phase III studies found that high AFP is a prognostic factor for poor OS of patients with liver cancer after treatment with sorafenib. Wen et al. (2015) found that AFP combined with miRNA can be used as an early blood biomarker for the detection of HCC. A multicentre analysis showed that HCC patients with AFP > 1,000 ng/mL had a worse survival after major hepatectomy (Andreou et al., 2013). Feng et al. (2017) also confirmed that AFP is an independent risk factor for recurrence among HCC patients after orthotopic liver transplantation. Interestingly, it has been reported that in patients with chronic hepatitis C and advanced fibrosis, AFP ≥ 200 ng/ml has a 99% specificity for the diagnosis of HCC, but the sensitivity is ≤20% (Sterling et al., 2012). This finding may be related to the increase in AFP and factors other than HCC.

There are many different treatments for HCC patients and are mainly classified surgical treatment, conservative treatment with internal medicine and treatment between them. Surgical treatment, including hepatectomy, is often considered the recommended treatment for HCC patients who are in compliance with the guidelines (Shi et al., 2007). Conservative treatment with internal medicine is often used in patients with advanced HCC without surgical indications, and this treatment has a certain improvement effect on the survival and prognosis of patients (Cheng et al., 2016; Ikeda et al., 2017). Considering the high cost of surgery and postoperative complications, as well as the poor efficacy of conservative medical treatment, therapeutic methods between medical and surgical treatment, such as radiofrequency ablation (Fang et al., 2014), are also being developed. According to our results, compared with other treatment methods, the selected surgical treatment of HCC patients resulted in a better survival rate and supports the view that surgery is the best treatment method for eligible patients.

Based on these meaningful variables, we incorporated them into the equation to construct the prognostic scoring model. To verify the predictive power of the model, we plotted the ROC curve, AUC at 1, 3 and 5 years are 0.797 (95% CI [0.781–0.813]), 0.762 (95% CI [0.742–0.782]) and 0.758 (95% CI [0.727–0.789]), suggested that the model has a good discrimination ability for predicting survival. Next, according to the X-tile software, we screened out the optimal cut-off value, and based on the patient’s OS, the patients were divided into a low-risk group, mid-risk group and a high-risk group. The Kaplan–Meier curve indicates that the survival of patients in the low-risk group was significantly higher than that in the high-risk group, indicating that this model has certain practicability. In addition, we also validated the model in the validation group. The results suggest that the performance of the model was also very stable in the validation group.

Previous prediction models for HCC have focused on patients with HCC of all ages or patients with early stage HCC (Wan et al., 2017; Chen et al., 2019). However, prognostic models based on a large number of elderly patients with HCC are rarely reported. Zhang et al. (2017) developed a prognostic model based on 423 HCC patients who were younger than 40 years old and incorporated clinically relevant factors such as neutrophil-lymphocyte ratio and HbeAg, but they did not validate their model. In contrast, despite the lack of some clinically relevant factors, our model included a large number of elderly HCC patients and was validated in the validation group, which included 1,076 patients. The results suggest that our model has a good predictive ability.

However, there are still some limitations to our study. Due to the lack of information in the database, we did not include the cause of HCC. Common causes of HCC include viral hepatitis and alcoholic hepatitis, as well as non-alcoholic hepatitis, which is now increasingly prevalent. The treatment and prognosis of HCC from different causes may also differ (Sangro et al., 2013; Mazzaferro et al., 2006; Kawada et al., 2009; Lee et al., 2019). Because of the lack of related data, the treatment options and risk of death selected by the patient cannot be assessed. In addition, we lack information on clinical indicators other than AFP. More indicators included in the study could have helped to identify clinical indicators with high specificity and sensitivity in elderly HCC patients.

## Conclusion

In conclusion, based on a large population, we constructed a prognostic scoring model for predicting survival in elderly HCC patients. The model has good discriminating ability and practicability, and may provide a reference for clinicians for the preoperative and postoperative evaluation of elderly HCC patients.

## Supplemental Information

10.7717/peerj.8497/supp-1 Supplemental Information 1 Raw data.

## Additional Information and Declarations

### Data Availability

The following information was supplied regarding data availability: Raw data is available at the SEER database and the Supplemental File. The raw measurements are available in Tables 1 and 2.
